# Supplementary material for: Transcriptome and Biochemical Analyses of a Chlorophyll-Deficient Bud Mutant of Tea Plant (Camellia sinensis)
Source: Int J Mol Sci. 2023 Oct 11;24(20):15070. doi: 10.3390/ijms242015070 (PMC10606798; doi:10.3390/ijms242015070)
Supplement: Supplementary file 1 [file ijms-24-15070-s001.zip › ijms-2633676-supplementary.pdf]

Table S1. Evaluation of transcriptome sequencing data.

Groups	Total Reads	Clean Reads	Clean Bases	GC(%)	Q20(%)	Total Mapped Reads	Ratio(%)
HY1	40836862	40471570	6.07G	43.86	97.26	35916783	88.75%
HY2	48759820	48254254	7.24G	44.59	97.54	43399693	89.94%
HY3	42106094	41559508	6.23G	44.51	97.58	37087698	89.24%
LY1	42657798	42085398	6.31G	44.42	97.62	37286420	88.60%
LY2	46700974	46025798	6.9G	44.35	97.71	40966016	89.01%
LY3	46251124	45422760	6.81G	44.85	97.64	40707219	89.62%

Table S2. Primer information.

Primer name	Sequence (5′-3′)
GAD-F	TGAATGGAGGGTGGGTCTCA
GAD-R	CGGACGTATCGAGAAGCGAA
ANS-F	GATGACTACAGTGGCTGCCC
ANS-R	AAGTATGTTGCCGATCCCCG
AAH-F	CTCCATGACGACCCGAAACT
AAH-R	AAGTAATTGGCATCTTTCTGTCTG
TASG-F	GGAGAGAGATGTGGTGCAGAC
TASG-R	ATGACTCCTGCAATGCCCTC
DFR-F	GGGATTTGTGTGCCGAAGC
DFR-R	TTGGGGTTCAAAGCTCCACA
GDHA-F	GAGTGAATCGTGTTGCACGG
GDHA-R	ATTCTTCAGCGGCAAAAATGC
CLH2-F	TAGGCCATGCCAAAACCCAA
CLH2-R	GAATGAGGGACATAGGTGAGGA
SGR-F	TCTTCAAAGCCATCCCCACC
SGR-R	AGTGGGAGAGGGTGTAGGTC
GS2-F	CTGGAACGGTGCAGGATGC
GS2-R	GCCCCACGCGGATTGAACA
CAO-F	GCAGACACTCAACAGCTCCT
CAO-R	GCATTGTCGGGTAGGTTCCA
POR-F	ATGGTGCCAAGGCCTACAAA
POR-R	AGCTTGGCTCACTTACCACC
GUN4-F	CAGCACAGAAGAGGGGCTAT
GUN4-R	CCCTCAGGTGTGTCATCACT
CHLH-F	CACCCTTTGGCCCCATGTAT
CHLH-R	CTCAACTGGCCCCGAGAAAT
CHLI-F	AGGGCAGAGTTATGGGAGGA
CHLI-R	CTGTTCTTGGGCAGGACTGA
CHLD-F	CGTTGTCGGACAGGATGCTA
CHLD-R	CATCTTCCCACTCTTCCGGG
Actin-F	GCCATATTTGATTGGAATGG
Actin-R	GGTGCCACAACCTTGATCTT

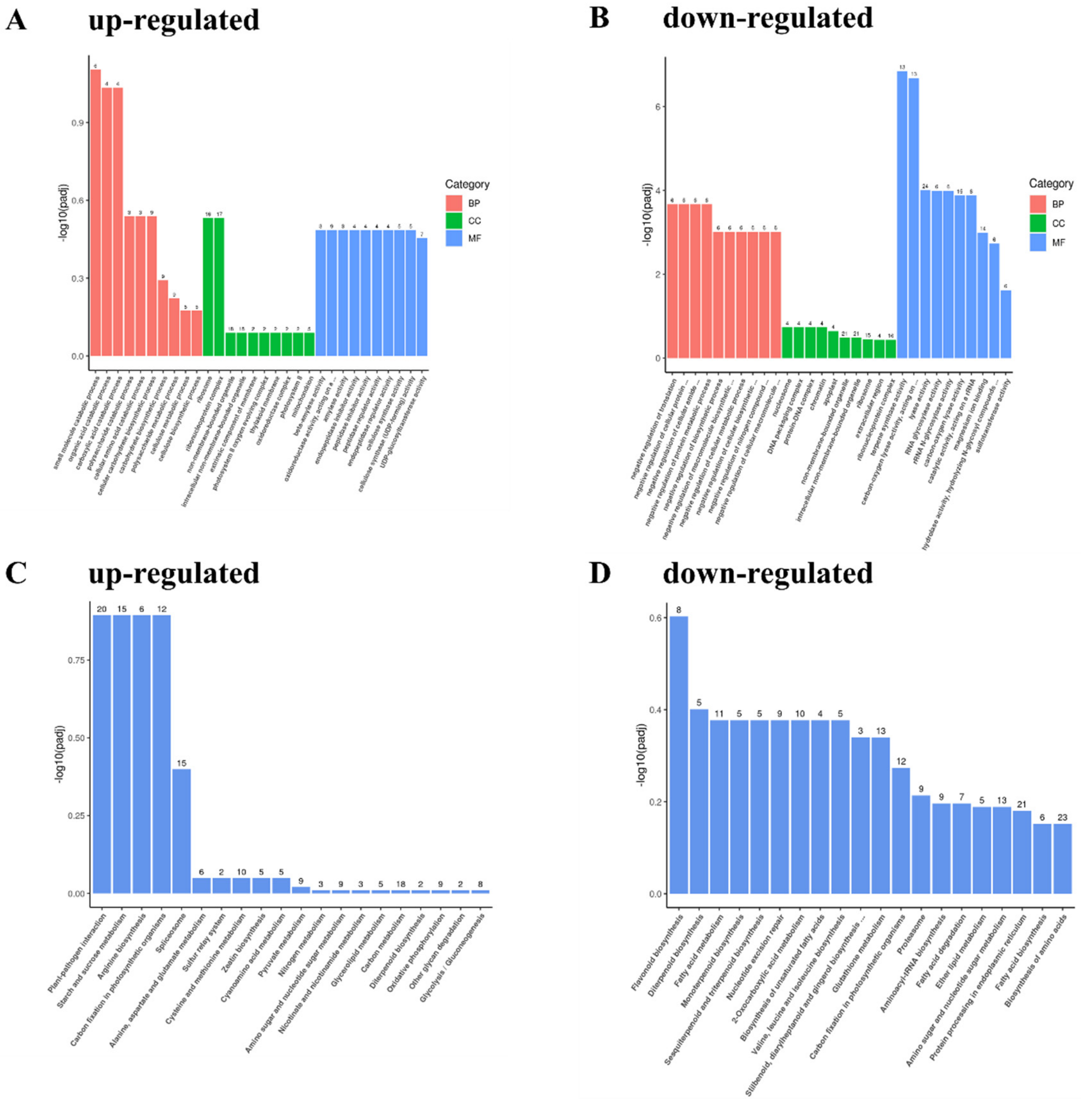

Figure S1. GO and KEGG enrichment analysis of DEGs between HY and LY. (A)-(B) Bar graph showing the top 30 enriched GO classifications of up- and down-regulated DEGs in three (BP, CC, MF) categories. BP, biological process; CC, cellular component; MF, molecular function; (C)-(D) Bar graph showing the top 20 enriched pathways of up- and down-regulated DEGs in KEGG analysis.

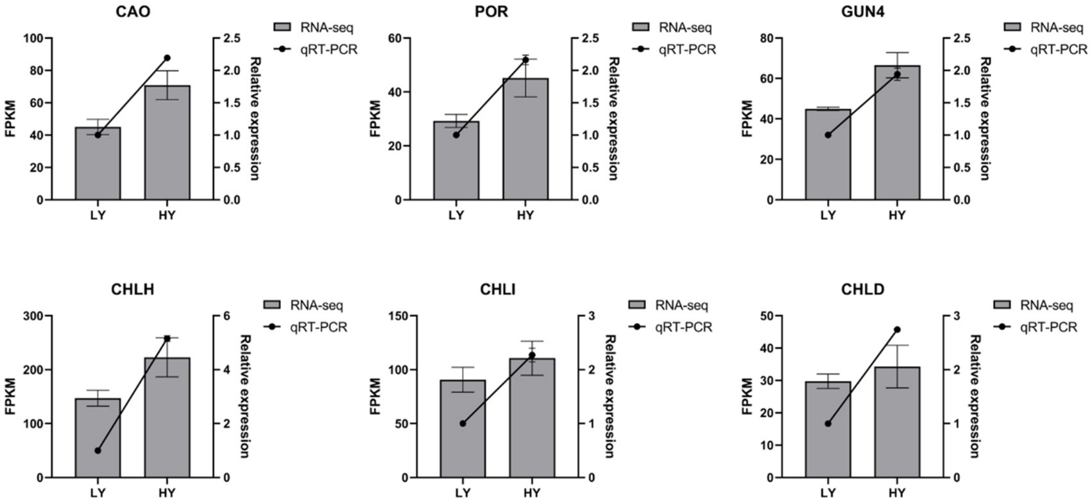

Figure S2. qRT-PCR validation of the six candidate DEGs related to chlorophyll biosynthesis. CAO, Chlorophyllide a oxygenase; POR, NADPH-protochlorophyllide oxidoreductase; GUN4, Genomes uncoupled 4; CHLH, Mg chelatase H subunit; CHLI, Mg chelatase I subunit ; CHLD, Mg chelatase D subunit.

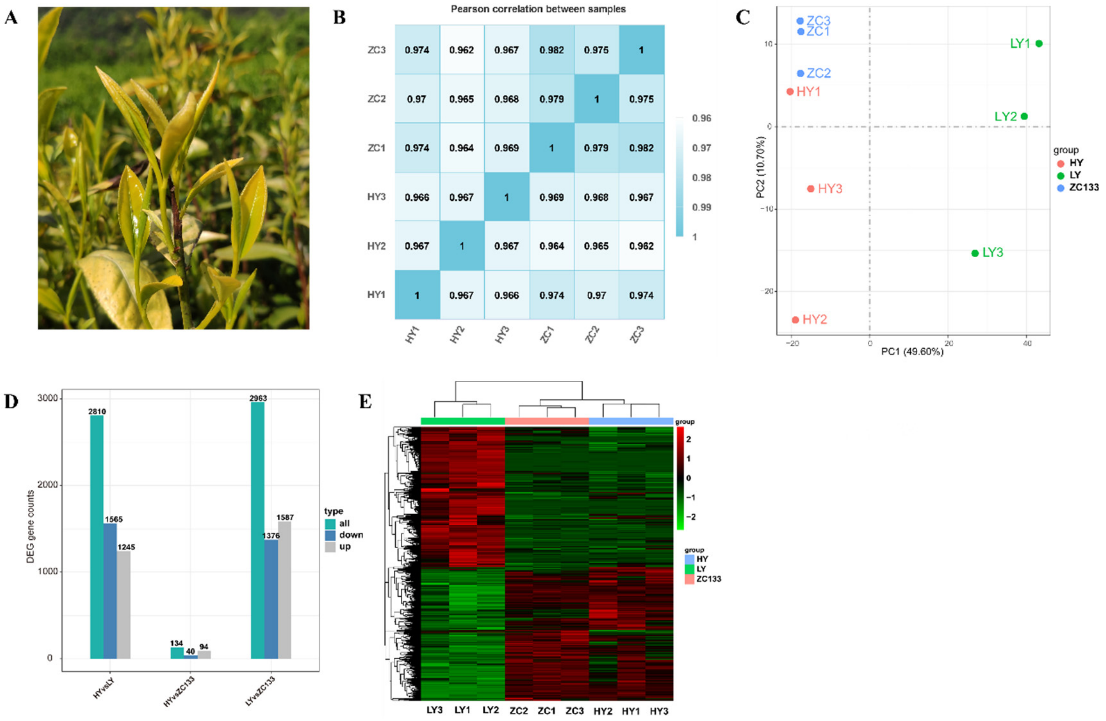

Figure S3. (A) Plant phenotype of 'Zhongcha 133' (captured on 11 April 2022); (B) Heatmap showing Pearson correlate coefficients of gene expression among different samples pairwise; (C) Principal component analysis (PCA) of the sequencing samples based on gene expression levels using their FPKM values. (D) Bar graph showing the number of total, up- and down-regulated DEGs between pairwise samples in different comparisons; (E) Cluster heat map of DEGs. The FPKM values of each gene expression levels were normalized with Z-score method. The redder color represents the higher expression levels and greener color represents lower levels. ZC1, ZC133-1; ZC2, ZC133-2; ZC3, ZC133-3. All the analyses were based on three biological replicates within groups.
